# Alterations of Dynamic Regional Homogeneity in Trigeminal Neuralgia: A Resting-State fMRI Study

**DOI:** 10.3389/fneur.2019.01083

**Published:** 2019-10-09

**Authors:** Jianhao Yan, Meng Li, Shishun Fu, Guomin Li, Tianyue Wang, Yi Yin, Guihua Jiang, Jingzhi Lin, Wuming Li, Jin Fang, Junzhang Tian

**Affiliations:** ^1^The Second School of Clinical Medicine, Southern Medical University, Guangzhou, China; ^2^The Department of Medical Imaging, Guangdong Second Provincial General Hospital, Guangzhou, China; ^3^The Department of Neruosurgery, Guangdong Second Provincial General Hospital, Guangzhou, China

**Keywords:** trigeminal neuralgia, resting-state fMRI, dynamic regional homogeneity, pain duration, brain function

## Abstract

Accumulating evidence from neuroimaging studies has supported that chronic pain could induce changes in brain function. However, few studies have focused on the dynamic regional homogeneity (dReHo) of trigeminal neuralgia (TN). In this study, twenty-eight TN patients and 28 healthy controls (HC) were included. Based on the resting-state fMRI (rsfMRI), we detected abnormalities in dReHo in the TN patients. Patients with TN had decreased dReHo in the left middle temporal gyrus, superior parietal lobule, and precentral gyrus, and increased dReHo in the thalamus. Furthermore, the increase in dReHo in the thalamus was positively correlated with duration of TN (*r* = 0.485, *p* = 0.012). These results provide compelling evidence for abnormal resting-state brain activity in TN and suggest that the duration of TN may play a critical role in brain function.

## Introduction

Trigeminal neuralgia (TN) is a common disease of the nervous system that manifests as episodes of severe pain over a distributed area of one or more branches of the trigeminal nerve (1). TN has an annual incidence of four to five per 100,000, and it is estimated that one in every 15,000–20,000 people worldwide is affected by TN (2–4). Despite the potential personal and social burden of TN, its pathogenesis remains poorly understood.

Neuroimaging studies have shown that chronic pain (e.g., back pain, migraine, and fibromyalgia) causes changes in brain structure and function (5–7). Recent studies have shown that TN is associated with pain, attention, emotion, and structural changes in the brain (8–11). Obermann et al. used voxel-based morphometry (VBM) to compare the brain morphology between TN patients and healthy individuals and showed that TN patients had a reduction in the gray-matter volume within multiple brain regions, which was similar to that found in our previous study of TN gray-matter volume (8, 11). However, to the best of our knowledge, fewer studies have explored the resting-state regional-homogeneity (ReHo) changes in TN patients and have yielded inconsistent results (12–14). For example, a study by Yuan et al. showed an increased ReHo in the anterior cingulate gyrus, middle temporal gyrus, and superior frontal gyrus and a decreased ReHo in the insula and cerebellum of TN patients, compared with those of the control group (13). In contrast, Wang et al. showed an increased ReHo in the inferior temporal gyrus, thalamus, inferior parietal lobule, and precentral and postcentral gyri of TN patients and a decreased ReHo in the amygdala, parahippocampal, and cerebellum (12). Xiang et al. reported an increased ReHo in the inferior temporal gyrus, fusiform gyrus, middle temporal gyrus, superior frontal gyrus, and precentral gyrus of TN patients and no decrease in ReHo (14). As such, whether or not TN development affects spontaneous neural activity remains uncertain.

ReHo analysis is a form of data-driven resting-state functional magnetic resonance imaging (rsfMRI) that measures the temporal similarity of a given voxel to that of adjacent voxels and does require knowledge of the experimental design in advance (15, 16). Recently, dynamic ReHo (dReHo) has been used as a research indicator to provide a new perspective for abnormal brain activity (16). A previous dReHo study using the sliding-window approach showed that brain regions with large fluctuations in dReHo are often functional centers in the brain (17). In addition, this research method has been extensively used in the study of depression, schizophrenia, and bipolar disorder (18–20). However, no relevant studies have reported in the changes of dReHo in TN patients.

To investigate the spontaneous neural activity in the brain of TN patients during the resting state, the present study measured the dReHo throughout the entire brain. Based on our previous findings, we hypothesized that TN patients have changes of dReHo compared to that of controls in some temporal, parietal regions. In addition, studies of different types of chronic pain have shown that the structural and/or functional changes in patients with pain are often associated with pain duration. Therefore, we also hypothesized that the duration of pain is related to abnormal dReHo.

## Materials and Methods

### Subjects

Permission to undertake this study was granted by the ethics committee of Guangdong Second Provincial General Hospital. In 2017, we recruited 28 TN patients. Each of the TN patients was screened according to the International Classification of Headache Disorders version III criteria (1) to confirm the diagnosis of TN. Prior to the examination, none of the patients had undergone any psychotherapy. The inclusion criteria for the patients were as follows: (i) age > 18 years; (ii) right-hand dominance; (iii) unilateral pain in the distribution of one or more branches (the ophthalmic [V1], the maxillary [V2], and the mandibular [V3]) of the trigeminal nerve; (iv) no psychiatric medications or substance abuse; (v) no MR imaging contraindications; and (vi) no head trauma or neurologic disorders. Exclusion criteria were: (i) patients with neural-associated diseases or chronic pain other than TN; (ii) patients with brain surgery history; (iii) contraindications to MRI.

Twenty-eight age- and gender-matched healthy controls (HC) were also recruited for this study. The inclusion criteria for healthy controls were as follows: (i) age > 18 years; (ii) right-hand dominance; (iii) no psychiatric medications or substance abuse; and (iv) no MR imaging contraindications. Written informed consent was obtained from each subject.

### Assessment of Mental Status

Before undergoing resting-state MRI, all TN patients were screened for International Classification of Headache Disorders-III and with visual-analog scales (VAS) in order to estimate the intensity and frequency of the symptoms. In addition, emotional assessments were conducted for all participants, via the self-rating anxiety scale (SAS) (21) and the self-rating depression scale (SDS) (22).

### Data Acquisition

The MR imaging data was acquired on a 3.0 T Philips Ingenia MR scanner using a 32-channel head coil at the department of Medical Imaging in Guangdong Second Provincial General Hospital. The resting-state fMRI data were acquired using gradient echo-planar imaging (EPI) with the following parameters: repetition time (TR)/echo time (TE) = 2,000 ms/30 ms; matrix = 64 × 64; field-of-view (FOV) = 230 × 230 mm; flip angle (FA) = 90; slice thickness = 3.6 mm, 0.6-mm gap; interleaved scanning; 38 transverse slices; 240 volumes; each volume was aligned along the anterior–posterior commissure. T1-weighted 3D high resolution brain structural images were obtained using a fast field echo (FFE) pulse sequence with TR/TE = 7.9/3.6 ms, matrix = 256 × 256, (FOV) = 256 × 256 mm, FA = 8°, slice thickness = 1.0 mm, and 186 sagittal slices.

### Resting-State fMRI Data Preprocessing

The preprocessing of the functional images was performed with the DPARSF 4.3 Advanced Edition (http://rfmri.org/DPARSF) and the SPM12 package (www.fil.ion.ucl.ac.uk/spm) based in MATLAB (Mathworks, Inc., Natick, MA, USA). First, for signal equilibration the first 10 volumes of each dataset were discarded, and the remaining data were processed with following steps: slice-timing correction, realignment and co-registration with the anatomical scan. Second, individual T1-weighted images were co-registered with the functional images, and then were segmented into gray matter, white matter and cerebrospinal fluid. Third, these functional images were then normalized into the Montreal Neurological Institute (MNI) space with a voxel size of 3 × 3 × 3 mm^3^. We eliminated the data of subjects with motion of more than 1.5-mm maximum displacement in any dimension and 1.5 degrees of angular motion during the entire fMRI scan. Fourth, linear-detrending processing was conducted to remove the linear-signal drift. Individual-level regression analysis was conducted to minimize the influence of head motion (Friston 24 model), white-matter signal noise, and cerebrospinal-fluid signal noise. A temporal band-pass filter (0.08–0.10 Hz) was applied to the data to remove the physical noise. Last, we performed spatial smoothing with an 8-mm full-width at-half-maximum (FWHM) kernel before performing the dReHo group analysis.

The dReHo calculation was as follows. The ReHo algorithm measured the voxel-wise short-distance functional connectivity with Kendall's coefficient of concordance (KCC) (15) using the following formula:

(1) $$\begin{array}{l} W=\frac{\sum_{i=1}^{N}{R_{i}}^{2}-N{\bar{R}}^{2}}{\frac{1}{12}K^{2}\left(N^{3}-N\right)}, \end{array}$$

where W is the KCC among the given voxels, N denotes the length of the time series, *K* = 27 is the size of the voxel cluster containing 3 × 3 × 3 adjacent voxels, R_i_ denotes the summation of the rankings of the BOLD signal amplitude of all K voxels at the i^th^ time point, and R is the mean of R_i_.

First-level dynamic analyses were conducted as follows. To compute the dReHo for these data, the time course was segmented into 60-s Hamming windows (30 dynamics). By sliding the onset of each window by two dynamics (4 s), for a total of 101 overlapping windows in the first-level analysis, the dReHo was estimated by using the calculated standard deviation (SD) of the ReHo through the windows at each voxel, yielding a set of ReHo maps for each participant.

Group-level dynamic analyses were completed as follows. A two-sample *t*-test was performed to test the difference in dReHo maps between the TN patients and healthy controls at each voxel (23, 24) with head-motion parameters (mean FD Jenkinson values), age, and sex as covariates. Correction for multiple comparisons was performed with false discovery rate (FDR) theory at the cluster level (*p* < 0.05, FDR correction).

In addition, we conducted analyses to test the association between the clinical indicators (i.e., pain duration, SAS, and SDS) of TN and dReHo within the TN group. We extracted the mean signal of each dynamic and compared the ReHo variability of the thalamus between TN patients and HCs (**Figure 2**). The correlation analysis was performed with the SPSS software with a signifcance threshold of *p* < 0.05 (uncorrected).

## Results

### Demographic and Clinical Characteristics

The demographic and clinical data are summarized in Table 1. No significant differences were observed between the TN and HC groups in terms of age, gender, or education (*p* > 0.05). In addition, SAS and SDS also showed no significant differences between these two groups. The average duration of pain in the TN group was 4.45 years.

**Table 1:** Demographic characteristics of the trigeminal neuralgia (TN) patients and the healthy controls (HC).

**Characteristic**	**TN (*n* = 28)**	**HC (*n* = 28)**	***T*-value**	***P*-value**
Age (year)	37.4 ± 9.0	40.3 ± 10.3	−1.01	0.32
Sex (male/female)	14/14	14/14		1
Education (year)	11.8 ± 3.2	10.5 ± 4.3	1.20	0.24
Pain duration (year)	4.5 ± 13.3	NA	–	–
Rating of clinical pain	8.9 ± 1.6	NA	–	–
SAS	36.1 ± 8.1	38.2 ± 6.0	−0.97	0.34
SDS	38.1 ± 9.5	39.1 ± 8.1	−0.39	0.70

*SAS, self-rating anxiety scale (SAS); SDS, self-rating depression scale; NA, not applicable*.

### dReHo Analysis

**Figure 1:**
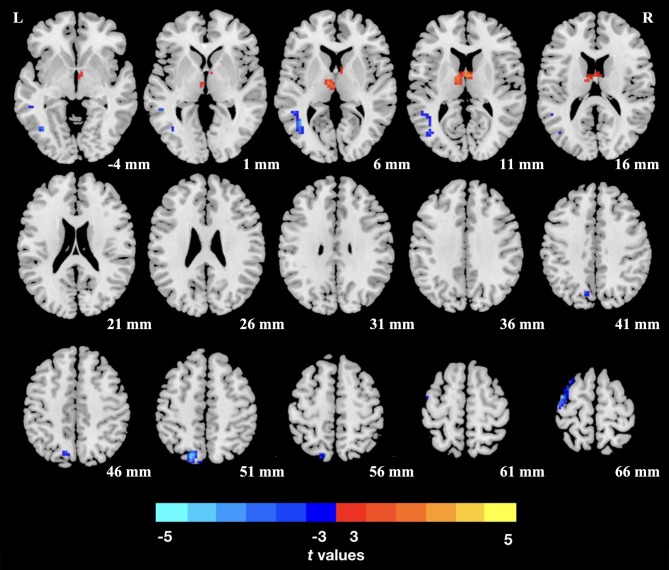
Group differences of dReHo variability were revealed by two-sample *t*-test. Clusters color-coded in blue (red) indicate significantly decreased (increased) dReHo in the trigeminal neuralgia (TN) patients. L (R), left (right) hemisphere.

The TN patients exhibited an increased dReHo (more variability) in the thalamus. We also found a decreased dReHo (less variability) in the left middle temporal gyrus (MTG), superior parietal lobule (SPL), and precentral gyrus (PCG; Table 2 and Figure 1).

**Table 2:** Brain regions showing significantly changed dynamic regional homogeneity in the trigeminal neuralgia (TN) patients compared to the healthy controls (HC).

**Brain region**	**Cluster size**	**MNI coordinates**			**AAL**	**Brodmann's area**	**Peak *T*-value**
		**X**	**Y**	**Z**			
L MTG	85	−42	−60	9	Temporal_Mid_L	39	−5.26
Thalamus	84	3	−3	12	Thalamus_L	–	5.16
L SPL	60	−15	−72	51	Parietal_Sup_L	7	−5.78
L PCG	41	−39	−6	66	Precentral_L	6	−5.81

*All regions listed are statistically significant at p < 0.05, FDR corrected*.

*MNI, Montreal Neurological Institute; AAL, Anatomical Automatic Labeling; L, left hemisphere; MTG, middle temporal gyrus; SPL, superior parietal lobule; PCG, precentral gyrus*.

### Correlation Analysis

**Figure 2:**
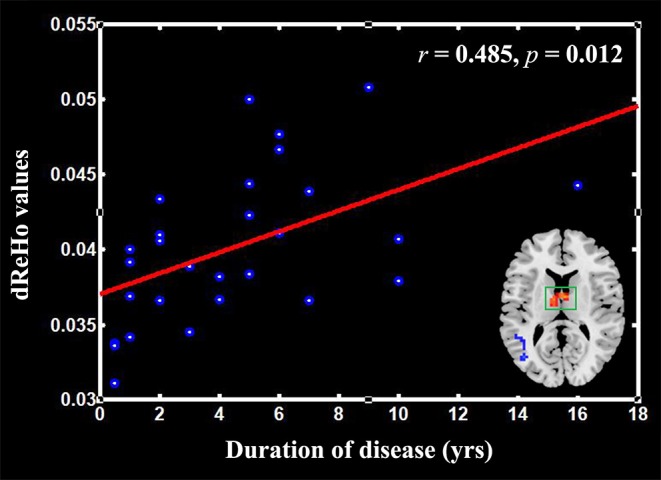
Scatter plots of the mean dReHo of the clusters in the thalamus positively correlated with the pain duration in the patients with trigeminal neuralgia (TN).

The correlation analysis revealed that the mean dReHo was significantly positively correlated with the duration of TN in the thalamus (*r* = 0.485, *p* = 0.012; Figure 2). However, no other positive or negative correlations were found between the mean dReHo values and disease duration in the other clusters listed in Table 2. In addition, we found no significant correlations between dReHo and pain duration, SAS or SDS.

## Discussion

In the present study, we used dReHo to explore the spontaneous neural activity in the brain of TN patients and found abnormal dReHo in TN patients compared to that of HCs. A decreased dReHo was found in the left temporal lobe, parietal lobe, and precentral gyrus. An increased dReHo was found in the thalamus of the TN patients. Importantly, the increased dReHo in the thalamus was related to the pain duration of the patients. This study highlights the abnormalities of pain-perception, pain-regulation, and motion-related systems in terms of altered spontaneous neural activity in corresponding brain regions (12, 25, 26). The associated brain regions that we found were altered in TN patients are known to be primarily involved in the pain-management process of the central nervous system.

The dReHo of the MTG was decreased in the present study. Many studies of chronic pain have shown pain-related activation of the temporal lobe, suggesting that MTG is involved in the perception of pain (27–29). For example, Freund et al. performed two experiments using fMRI. The tested subjects were required to distinguish between different degrees of thermal stimulations, and activations in the insula, parietal lobe, and temporal lobe were found (28). A study by Rottmann et al. evaluated the effects of low-frequency electrical stimulation on brain activation and showed that the test stimulation activated the insula, anterior cingulate cortex, superior temporal gyrus, and prefrontal cortex. Brain morphological studies have also confirmed changes in temporal cortical structure in patients with different pain disorders (30). Schmidt-Wilcke et al. used voxel-based morphometry (VBM) to study a reduction in gray-matter volumes in the left anterior cingulate gyrus and left temporo-insular region in patients with persistent idiopathic facial pain (31). One of our previous morphological studies on TN has shown a reduced gray matter volume in the middle temporal cortex (11). Combined with previous studies, our present findings might indicate that the temporal cortex is susceptible to chronic pain, and abnormalities in the MTG may be associated with chronic pain perception. In addition, the present study showed that the dReHo of the parietal cortex was reduced, and that both the MTG and parietal cortex (especially the angular gyrus) belonged to the default mode network (DMN) (32). The DMN is involved in internal processing, including autobiographic memory, self-reference, and stimulus-independent thoughts (32, 33). When the DMN is not in contact with the external environment, it is usually active (34). We observed a decreased ReHo in the temporal cortex and the parietal cortex, suggesting a maladaptation of brain activity caused by pain. It has been reported that the lateral parietal cortex is involved in pain prediction through meta-analysis, which may represent the second level of the pain-information-processing circuit that supports the active, conscious, and cognitive assessment of pain perception (7).

In addition, in the present study, an abnormal change in the ReHo value of the left precentral gyrus was found compared with that of HCs. The precentral gyrus is part of the primary motor cortex that reflects the sensory pain response, inhibition of maxillary movement, and facial muscle tension (35). Even simple and painless exercise can cause painful episodes in TN patients (12). Therefore, limiting facial movement can reduce pain. A change in the dReHo value of the precentral gyri suggested that there is a local synchronization of brain activity and pain regulation in TN patients. Thus, we hypothesize that the primary motor cortex inhibits the pain response of the trigeminal nerve and inhibits the maxillary tension to relieve pain.

In addition, increased dReHo was mainly located in the thalamus, which is consistent with previous findings (12, 36). A previous study showed increased ReHo in the thalamus of TN patients and a low N-acetylaspartate/creatine ratio in the thalamus on the affected side (37). Moreover, brain structural examinations showed that the thalamic volume of TN patients was higher than that of controls in the previous study (36). Thus, we speculate that the increased dReHo may reflect a persistent injury-induced input caused by pain, which is closely related to the symptoms of TN. We also found that the mean dReHo value of the thalamus was positively correlated with the duration of disease; the longer the pain lasted, the higher the dReHo of the thalamus, suggesting that chronic pain alters spontaneous brain activity in function.

This study has some limitations. First, the cross-sectional design of this study was not able to study the causal relationship between the functional abnormalities and TN development. Second, this study only observed correlations between the imaging and duration of TN, which may be due to the small sample size of the TN group. Thus, further studies with larger sample sizes will be necessary to verify the findings of our present study. Third, uneven distribution of the affected side (9 left/19 right) may contribute to the left-lateralization of dReHo, which may indicate neuroadaptation or possible compensatory changes. Future studies should take the impact of the affected side in consideration. Lastly, all TN patients of this study consumed painkillers and, therefore, we cannot rule out the possible confounding effects of drugs on the ReHo analysis.

In brief, this study used resting-state analysis to measure the changes of dReHo in TN patients, which were found to be mainly in the thalamus and some areas of the temporal lobe and parietal lobe. These brain areas are primarily involved in pain perception and regulation. The present study also showed that the increased dReHo in the thalamus was associated with increased pain duration in TN patients. These results provide important information for the limited studies of brain functional changes in TN patients.

## Data Availability Statement

The datasets generated for this study are available on request to the corresponding author.

## Ethics Statement

The studies involving human participants were reviewed and approved by Ethics committee of Guangdong Second Provincial General Hospital. The patients/participants provided their written informed consent to participate in this study.

## Author Contributions

JY designed the experiment. JY and ML carried out the experiment. GL and YY collected and sorted out the data. SF, TW, GJ, JL, WL, and JF helped on data management and processing. JY, ML, and JT wrote the manuscript.

### Conflict of Interest

The authors declare that the research was conducted in the absence of any commercial or financial relationships that could be construed as a potential conflict of interest.

## References

[1] Headache Classification Committee of the International Headache S The international classification of headache disorders, 3rd edition (beta version). Cephalalgia. (2013) 33:629–808. 10.1177/0333102413485658 23771276

[2] Katusic S Williams DB Beard CM Bergstralh EJ Kurland LT. Epidemiology and clinical features of idiopathic trigeminal neuralgia and glossopharyngeal neuralgia: similarities and differences, Rochester, Minnesota, 1945–1984. Neuroepidemiology. (1991) 10:276–81. 10.1159/000110284 1798430

[3] Gronseth G Cruccu G Alksne J Argoff C Brainin M Burchiel K. Practice parameter: the diagnostic evaluation and treatment of trigeminal neuralgia (an evidence-based review): report of the Quality Standards Subcommittee of the American Academy of Neurology and the European Federation of Neurological Societies. Neurology. (2008) 71:1183–90. 10.1212/01.wnl.0000326598.83183.04 18716236

[4] Mueller D Obermann M Yoon MS Poitz F Hansen N Katsarava Z. Prevalence of trigeminal neuralgia and persistent idiopathic facial pain: a population-based study. Cephalalgia. (2011) 31:1542–8. 10.1177/0333102411424619 21960648

[5] Simons LE Moulton EA Linnman C Carpino E Becerra L Borsook D. The human amygdala and pain: evidence from neuroimaging. Hum Brain Mapp. (2014) 35:527–38. 10.1002/hbm.22199 23097300 PMC3920543

[6] Kregel J Meeus M Malfliet A Dolphens M Danneels L Nijs J. Structural and functional brain abnormalities in chronic low back pain: a systematic review. Semin Arthritis Rheum. (2015) 45:229–37. 10.1016/j.semarthrit.2015.05.002 26092329

[7] Palermo S Benedetti F Costa T Amanzio M. Pain anticipation: an activation likelihood estimation meta-analysis of brain imaging studies. Hum Brain Mapp. (2015) 36:1648–61. 10.1002/hbm.22727 25529840 PMC6869158

[8] Obermann M Rodriguez-Raecke R Naegel S Holle D Mueller D Yoon M Katsarava Z. Gray matter volume reduction reflects chronic pain in trigeminal neuralgia. Neuroimage. (2013) 74:352–8. 10.1016/j.neuroimage.2013.02.029 23485849

[9] Parise M Kubo TT Doring TM Tukamoto G Vincent M Gasparetto EL. Cuneus and fusiform cortices thickness is reduced in trigeminal neuralgia. J Headache Pain. (2014) 15:17. 10.1186/1129-2377-15-17 24661349 PMC3997919

[10] Parise M Acioly MA Vincent M Gasparetto EL Decision-making in classic trigeminal neuralgia concurrent with a pontine cavernous malformation: causal or coincidental association? Neurocirugia. (2015) 26:90–4. 10.1016/j.neucir.2014.09.003 25450011

[11] Li M Yan J Li S Wang T Zhan W Wen H. Reduced volume of gray matter in patients with trigeminal neuralgia. Brain Imaging Behav. (2017) 11:486–92. 10.1007/s11682-016-9529-2 26899433

[12] Wang Y Zhang X Guan Q Wan L Yi Y Liu CF. Altered regional homogeneity of spontaneous brain activity in idiopathic trigeminal neuralgia. Neuropsychiatr Dis Treat. (2015) 11:2659–66. 10.2147/NDT.S94877 26508861 PMC4610767

[13] Yuan J Cao S Huang Y Zhang Y Xie P Zhang Y. Altered spontaneous brain activity in patients with idiopathic trigeminal neuralgia: a resting-state functional MRI study. Clin J Pain. (2018) 34:600–9. 10.1097/AJP.0000000000000578 29252869 PMC5999362

[14] Xiang CQ Liu WF Xu QH Su TS Yong-Qiang MY Jiang NJPP. Altered spontaneous brain activity in patients with classical trigeminal neuralgia using regional homogeneity: a resting-state functional MRI *Study*. Pain Pract. (2019) 19:397–406. 10.1111/papr.12753 30536573

[15] Zang Y Jiang T Lu Y He Y Tian L. Regional homogeneity approach to fMRI data analysis. Neuroimage. (2004) 22:394–400. 10.1016/j.neuroimage.2003.12.030 15110032

[16] Zuo XN Xu T Jiang L Yang Z Cao XY Milham MP. Toward reliable characterization of functional homogeneity in the human brain: preprocessing, scan duration, imaging resolution and computational space. Neuroimage. (2013) 65:374–86. 10.1016/j.neuroimage.2012.10.017 23085497 PMC3609711

[17] Hutchison RM Womelsdorf T Allen EA Bandettini PA Calhoun VD Chang C. Dynamic functional connectivity: promise, issues, and interpretations. Neuroimage. (2013) 80:360–78. 10.1016/j.neuroimage.2013.05.079 23707587 PMC3807588

[18] Damaraju E Allen EA Belger A Ford JM McEwen S Calhoun VD. Dynamic functional connectivity analysis reveals transient states of dysconnectivity in schizophrenia. Neuroimage Clin. (2014) 5:298–308. 10.1016/j.nicl.2014.07.003 25161896 PMC4141977

[19] Rashid B Damaraju E Pearlson GD Calhoun VD. Dynamic connectivity states estimated from resting fMRI Identify differences among Schizophrenia, bipolar disorder, and healthy control subjects. Front Hum Neurosci. (2014) 8:897. 10.3389/fnhum.2014.00897 25426048 PMC4224100

[20] Qiu L Xia M Cheng B Yuan L Kuang W Bi F Abnormal dynamic functional connectivity of amygdalar subregions in untreated patients with first-episode major depressive disorder. J Psychiatry Neurosci. (2018) 43:262–72. 10.1503/jpn.170112 29947609 PMC6019355

[21] Zung WW. A rating instrument for anxiety disorders. Psychosomatics. (1971) 12:371–9. 10.1016/S0033-3182(71)71479-0 5172928

[22] Zung WW. A self-rating depression scale. Arch Gen Psychiatr. (1965) 12:63–70. 10.1001/archpsyc.1965.01720310065008 14221692

[23] Jenkinson M Bannister P Brady M Smith S. Improved optimization for the robust and accurate linear registration and motion correction of brain images. Neuroimage. (2002) 17:825–41. 10.1006/nimg.2002.1132 12377157

[24] Yan CG Craddock RC Zuo XN Zang YF Milham MP. Standardizing the intrinsic brain: towards robust measurement of inter-individual variation in 1000 functional connectomes. Neuroimage. (2013) 80:246–62. 10.1016/j.neuroimage.2013.04.081 23631983 PMC4074397

[25] Schweinhardt P Bushnell MC. Pain imaging in health and disease–how far have we come? J Clin Invest. (2010) 120:3788–97. 10.1172/JCI43498 21041961 PMC2964988

[26] May A. Structural brain imaging: a window into chronic pain. Neuroscientist. (2011) 17:209–20. 10.1177/1073858410396220 21489967

[27] Buchsbaum BR Hickok G Humphries CJCS Role of left posterior superior temporal gyrus in phonological processing for speech perception and *production*. Cognit Sci Multidiscipl J. (2001) 25:663–78. 10.1207/s15516709cog2505_2

[28] Freund W Klug R Weber F Stuber G Schmitz B Wunderlich AP. Perception and suppression of thermally induced pain: a fMRI study. Somatosens Mot Res. (2009) 26:1–10. 10.1080/08990220902738243 19283551

[29] Smallwood RF Laird AR Ramage AE Parkinson AL Lewis J Robin DA. Structural brain anomalies and chronic pain: a quantitative meta-analysis of gray matter volume. J Pain. (2013) 14:663–75. 10.1016/j.jpain.2013.03.001 23685185 PMC4827858

[30] Rottmann S Jung K Vohn R Ellrich J. Long-term depression of pain-related cerebral activation in healthy man: an fMRI study. Eur J Pain. (2010) 14:615–24. 10.1016/j.ejpain.2009.10.006 19896873

[31] Schmidt-Wilcke T Hierlmeier S Leinisch E. Altered regional brain morphology in patients with chronic facial pain. Headache. (2010) 50:1278–85. 10.1111/j.1526-4610.2010.01637.x 20236343

[32] Raichle ME. The brain's default mode network. Annu Rev Neurosci. (2015) 38:433–47. 10.1146/annurev-neuro-071013-014030 25938726

[33] Andrews-Hanna JR Smallwood J Spreng RN. The default network and self-generated thought: component processes, dynamic control, and clinical relevance. Ann N Y Acad Sci. (2014) 1316:29–52. 10.1111/nyas.12360 24502540 PMC4039623

[34] Kucyi A Moayedi M Weissman-Fogel I Goldberg MB Freeman BV Davis KD. Enhanced medial prefrontal-default mode network functional connectivity in chronic pain and its association with pain rumination. J Neurosci. (2014) 34:3969–75. 10.1523/JNEUROSCI.5055-13.2014 24623774 PMC6705280

[35] Ellingson LD Shields MR Stegner AJ Cook DB. Physical activity, sustained sedentary behavior, and pain modulation in women with fibromyalgia. J Pain. (2012) 13:195–206. 10.1016/j.jpain.2011.11.001 22245361 PMC3272134

[36] Desouza DD Moayedi M Chen DQ Davis KD Hodaie M. Sensorimotor and pain modulation brain abnormalities in trigeminal neuralgia: a paroxysmal, sensory-triggered neuropathic pain. PLoS ONE. (2013) 8:e66340. 10.1371/journal.pone.0066340 23823184 PMC3688879

[37] Wang Y Li D Bao F Ma S Guo C Jin C. Thalamic metabolic alterations with cognitive dysfunction in idiopathic trigeminal neuralgia: a multivoxel spectroscopy study. Neuroradiology. (2014) 56:685–93. 10.1007/s00234-014-1376-5 24820951

